# The Impact of Computational Uncertainties on the Enantioselectivity Predictions: A Microkinetic Modeling of Ketone Transfer Hydrogenation with a Noyori‐type Mn‐diamine Catalyst

**DOI:** 10.1002/cctc.202100341

**Published:** 2021-06-10

**Authors:** Annika M. Krieger, Evgeny A. Pidko

**Affiliations:** ^1^ Inorganic Systems Engineering Department of Chemical Engineering Faculty of Applied Sciences Delft University of Technology Van der Maasweg 9 2629 HZ Delft The Netherlands

**Keywords:** density functional theory, transfer hydrogenation, enantioselectivity, Noyori-type catalysis, microkinetic modeling

## Abstract

Selectivity control is one of the most important functions of a catalyst. In asymmetric catalysis the enantiomeric excess (e.e.) is a property of major interest, with a lot of effort dedicated to developing the most enantioselective catalyst, understanding the origin of selectivity, and predicting stereoselectivity. Herein, we investigate the relationship between predicted selectivity and the uncertainties in the computed energetics of the catalytic reaction mechanism obtained by DFT calculations in a case study of catalytic asymmetric transfer hydrogenation (ATH) of ketones with an Mn‐diamine catalyst. Data obtained from our analysis of DFT data by microkinetic modeling is compared to results from experiment. We discuss the limitations of the conventional reductionist approach of e.e. estimation from assessing the enantiodetermining steps only. Our analysis shows that the energetics of other reaction steps in the reaction mechanism have a substantial impact on the predicted reaction selectivity. The uncertainty of DFT calculations within the commonly accepted energy ranges of chemical accuracy may reverse the predicted e.e. with the non‐enantiodetermining steps contributing to e.e. deviations of up to 25 %.

## Graphical abstract

**Microkinetic modeling**: The ratio of the R‐ to S‐conformer in asymmetric catalysis depends on the ratio of the rates for the respective competing reaction channels. These are usually approximated by the free energy difference in the barriers of the enantio‐determining step. Herein we combined DFT calculations with microkinetic modeling of a model reaction of ketone transfer hydrogenation by Mn(I)‐NN catalyst to quantitatively analyze the validity of this approximation and the role other steps of the catalytic mechanism play for the predicted enantioselectivity profiles.

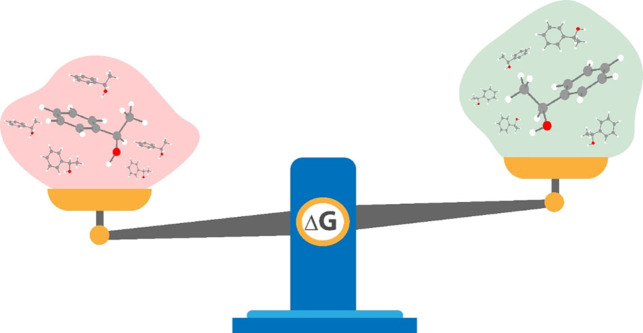

## Introduction

Asymmetric reduction catalysis is a powerful tool for the production of chiral compounds. Enantiomeric purity is desirable in the production of fine chemicals such as in the pharmaceutical and crop‐protection industry.^[1]^ Discovery of efficient enantioselective catalysts involves tedious work largely based on the trial and error approach. There is a long‐standing dream of rational design based on ab initio calculations.^[2]^ Substantial progress has been made in this direction in the recent years with the introduction of novel data‐rich computational screening approaches allowing for rapid evaluation of diverse reactivity descriptors for transition metal catalysts and correlating them with defined reactivity metrics.[^3^, ^4^, ^5^]

Despite great progress witnessed in the last decade[^6^, ^7^], computational elucidation of enantioselectivity still mostly follows synthesis instead of leading it.[^8^, ^9^, ^10^, ^11^, ^12^] Density functional theory (DFT) is currently the method of choice for computing mechanisms and energetics of competing reaction paths in homogeneous catalysis by transition metals.[^13^, ^14^, ^15^] Although the accuracy of modern computational methods is being constantly improved through the efforts of a large community of researchers, reaching the chemical accuracy of 5 kJ mol^−1^ is still a great challenge for practical electronic structure methods.[^16^, ^17^, ^18^]

Besides the accuracy of the computational method, the uncertainty regarding the reaction mechanism and the nature of the catalytic species can also affect the predictive power. Multi‐step mechanisms with relatively short‐lived intermediates are common in homogenous catalysis resulting in highly complex kinetic behavior. The enantioselectivity is determined by the relative rates of the two competing enantioselective paths. Given the high complexity of most catalytic mechanisms, in practice e.e.’s are commonly estimated by comparing only the computed barriers of the two enantiodetermining steps, while assuming that the other reaction steps in the reaction mechanism have only minor impact on the overall kinetics and the e.e. of the reaction.

**Scheme 1:**
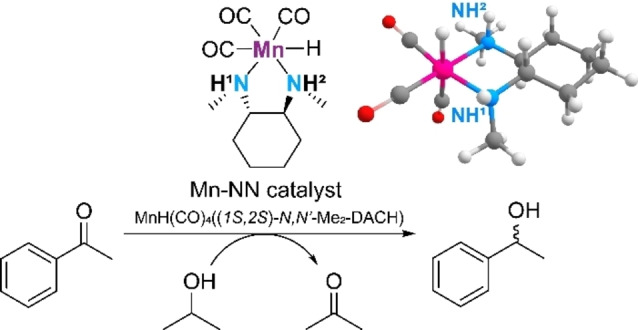
Mn‐diamine catalyst with reaction site NH1 and NH2 and the model reaction of acetophenone to phenylethanol heme Caption.

One way to tackle the mechanistic complexity of catalysis are ab initio kinetic models. Microkinetic models offer the possibility to investigate complete reaction pathways by calculating rate constants and modeling concentration profiles.^[19]^ Microkinetic models are especially useful for discriminating between competing reactions and showing the role of elementary steps.^[20]^ While DFT‐calculations provide valuable insight about the reactivity, reaction paths and intermediates, microkinetic models offer an opportunity to analyze deeper the behavior of the catalytic systems and the kinetic details of the reaction. More complex reaction processes can hence be considered, and enantioselective excess can be determined while accounting for the whole reaction network. In this paper we investigate how minor variations in the energetics of the reaction pathway affect the selectivity of the catalyst with transfer hydrogenation of acetophenone in isopropanol using cis‐Mn(N,N’‐dimethyl‐1,2‐cyclohexanediamine)(CO)_3_Br as a model (Scheme 1).^[21]^ The availability of the detailed experimental kinetic data and the intermediate values of e.e. obtained with this catalyst makes it an attractive system to study the predictive power of theoretical models. Here we carried out an extended mechanistic analysis by considering all 4 alternative reaction channels for ketone transfer hydrogenation by DFT calculations and construction of a microkinetic model.

Selective reduction of organic oxygenates and their derivatives such as ketones, carboxylic acid esters and nitriles is fundamental to the production of fragrances, pharmaceuticals and fine‐chemicals.[^22^, ^23^] In this context, asymmetric catalysis has led to exceptional advances in chemical synthesis.[^24^, ^25^] Besides, catalyst use is one of the most cost‐effective and environmentally responsible method to circumvent the need for stoichiometric reductants.[^26^, ^27^]

In recent years, significant progress has been made in the enantioselective (transfer) hydrogenation of polar C=O and C=N functionalities.[^28^, ^29^, ^30^, ^31^, ^32^, ^33^, ^34^] All these advances are aimed to achieve the highest possible yield and enantioselectivity under mild conditions. The asymmetric transfer hydrogenation of ketones by transition metal catalysts has been studied and the catalytic mechanism has been addressed by numerous computational and experimental works.[^31^, ^35^, ^36^] In the last decade substantial efforts were put in the development of new catalysts based on earth‐abundant elements. Several efficient enantioselective Mn(I)‐based reduction catalysts have been reported.[^8^, ^9^, ^37^] Transfer hydrogenation by acid‐base cooperative catalysts has been investigated both computationally and experimentally.[^38^, ^39^, ^40^, ^41^, ^42^, ^43^, ^44^, ^45^, ^46^, ^47^, ^48^] Evidence has been presented for both an inner‐ and outer‐sphere pathways.[^49^, ^50^]

Mn(I)‐based enantioselective catalysts are rare and usually underperform compared to their Ru or Rh‐based counterparts. Computational screening is an attractive approach to accelerate the development of such bio‐compatible and cheaper Mn‐based enantioselective catalysts. Herein, we employ a DFT‐based microkinetic modeling to explore how accurate e.e. predictions can be, assuming the chemical accuracy of DFT calculations and taking a kinetic model of the complete catalytic cycle into account for a simple chiral Mn‐diamine catalysts. We apply a multidimensional analysis to identify the factors contributing towards selectivity aside from the energy difference between the enantiodetermining steps.

## The Enantiomeric Excess

Enantiomeric excess is defined as the excess of the major enantiomer over the other[^51^, ^52^] (Eq. 1:

(1) $$e.e.=\frac{\left|c^{R}-\;c^{S}\right|}{c^{R}+\;c^{S}}$$

The ratio of the R‐ to S‐enantiomer is directly linked to the ratio of the rate constants for the two competing reaction channels for the conversion of the prochiral substance to the particular enantiomers (Eq. 2), which is in turn linked to the difference in apparent Gibbs free energies of activation of the two competing reaction paths of the diastereomeric transition state. Conventionally the intrinsic free energy of the enantiodetermining step is employed to reduce the kinetic complexity. Instead of the ratios of the overall rates, difference of activation free energies of the enantiodetermining steps are considered:

(2) $$\frac{c_{R}}{c_{S}}=\frac{k_{c_{R}}}{k_{c_{S}}}=e^{-\frac{{\Delta \Delta G}^{\neq }\;}{RT}}$$

Combing the two equations enables us to determine the e.e. from the difference in the transition state energy of the two enantiomers in the stereo selective step (Eq. 3.

(3) $$e.e.=\frac{\left|e^{-\frac{{\Delta \Delta G}^{\neq }\;}{RT}}-1\right|}{e^{-\frac{{\Delta \Delta G}^{\neq }\;}{RT}}+1}$$

**Figure 1:**
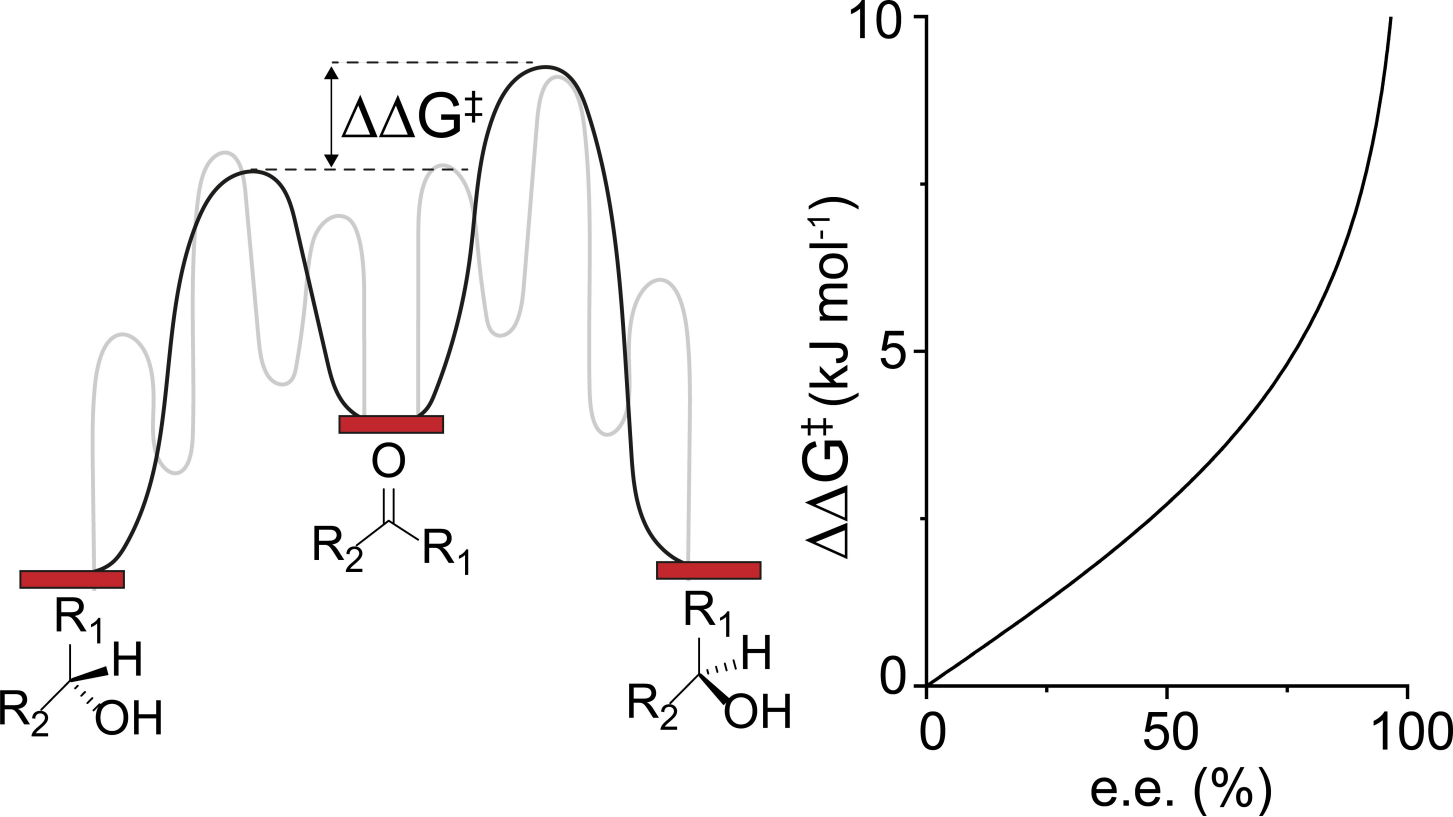
Relationship of the difference in the enantiodetermining transition state barrier and the enantiomeric excess assumed for the catalytic reaction. The black line indicates the two barriers for the enantiodetermining step, while the grey lines in the background represent the complete reaction network.

Therefore, the apparent barrier that is observed in experimental and theoretical investigation directly determines the value of our mathematical determined e.e. as shown in Figure 1.

This model does not account for the kinetic nature of the enantiomeric excess and its potential variations during the reaction. In practice, the complex reaction network contributes to the e.e.’s that is a function of conversion due to the interplay of forward and backward reactions.

## Results and Discussion

**Figure 2:**
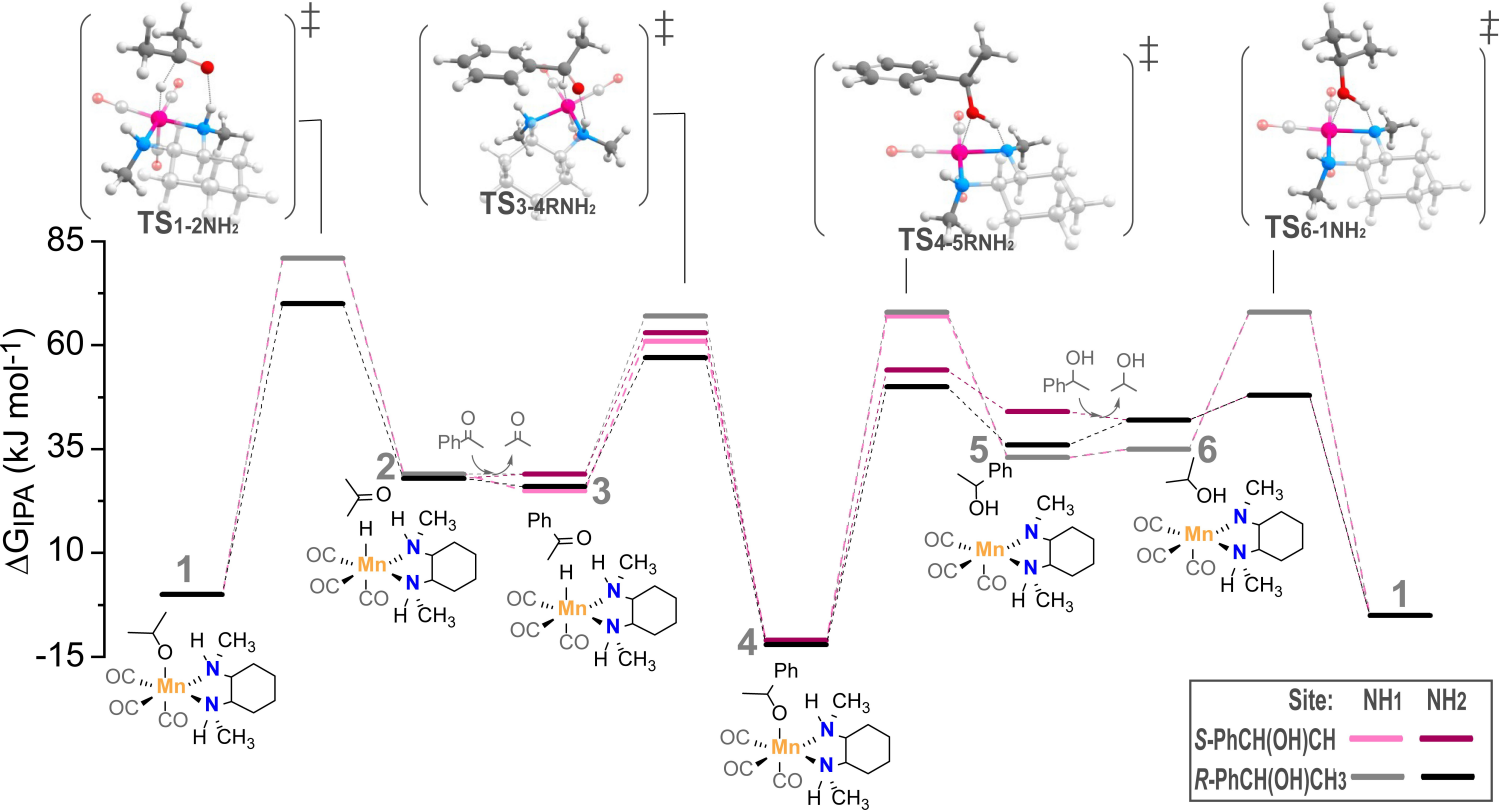
Standard Gibbs free energy diagram and the mechanism for the asymmetric transfer hydrogenation of acetophenone with iso‐propanol to R‐ and S‐phenylethanol at both NH‐reaction sites of Mn−N,N catalyst. Energies are given in kJ mol^−1^.

In this work we considered the asymmetric Mn‐diamine catalyst for the reduction of ketones. The hydrogenation of acetophenone to R‐ and S‐phenylethanol was investigated as a model reaction. Figure 2 presents the postulated reaction mechanism along with the DFT‐computed free energy diagram. The reaction can go along two channels distinguished as NH1 and NH2 here. Previous studies identified Mn(I)‐alkoxide species as the key reaction intermediates. The catalytic cycle starts with a Mn‐isopropoxide complex 1 that undergoes the β‐H elimination to produce 2 that is the Mn‐hydrido complex with a weakly bound acetone molecule. Acetone is in the next step replaced by acetophenone substrate, which aligns its carbonyl group with the Mn−H and N−H moieties of the catalyst (3). The hydride from the metal center is transferred to the ketone to form a stable alkoxide species 4. This reaction step is also the enantiodetermining step, which dictates the chirality of the produced alcohol. In the most endergonic reaction step, 1‐phenylethanol (PhCH(OH)CH_3_) is liberated, leading to the formation of the dehydrogenated Mn‐amido species 5. In the final step of the catalytic cycle, isopropanol reacts with the dehydrogenated catalyst to regenerate the initial Mn(I)‐isopropoxide state 1.

The enantiodetermining step in the lowest energy reaction pathway of the formation of R‐ to S‐phenylethanol shows a difference in Gibbs free energy of 6 kJ mol^−1^ (Figure 2). This difference translates with equation 3 to an e.e. of 79 %. The MKM model including all the reaction steps of the catalytic mechanism with the kinetic parameters directly computed from DFT predicts a similar e.e. of 77 %, which (coincidentally) is in a perfect agreement with the experimental values in the range 72–75 % (see Figure S1).

**Figure 3:**
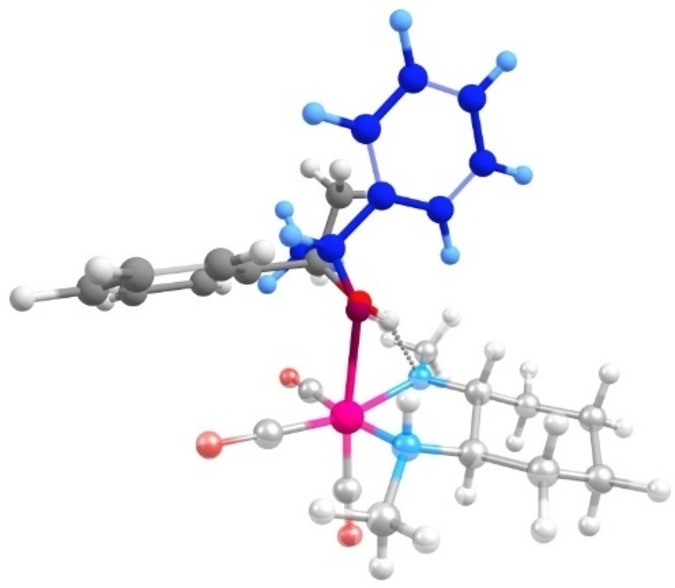
Overlay of two transition state structures from MD exhaustive conformational search, differing 5 kJ mol^−1^. The conformational difference is highlighted by the grey and blue structure.

The uncertainty of predicted energy barriers can be complex to quantify.[^53^, ^54^] Conventionally, energy uncertainty of 5 kJ mol^−1^ is considered as the chemical accuracy.[^16^, ^17^, ^18^] Such deviations can be brought about both by the method and model accuracy of the calculations. For the latter, minor conformational changes in the computed structures may give rise to such deviations. To check this hypothesis, we employed the exhaustive conformational transition state search method by Medvedev et al^[55]^ on the transition state of 4R to 5R proceeding through channel NH2. The conformations of the transition state were explored using the ab initio molecular dynamic simulations (see section S8 of the supplementary information). The lowest energy configurations extracted from the resulting trajectories were used as the input for the standard TS search. This approach revealed an alternative TS configuration (Figure 3) 5 kJ mol^−1^ above the original TS structure.

**Figure 4:**
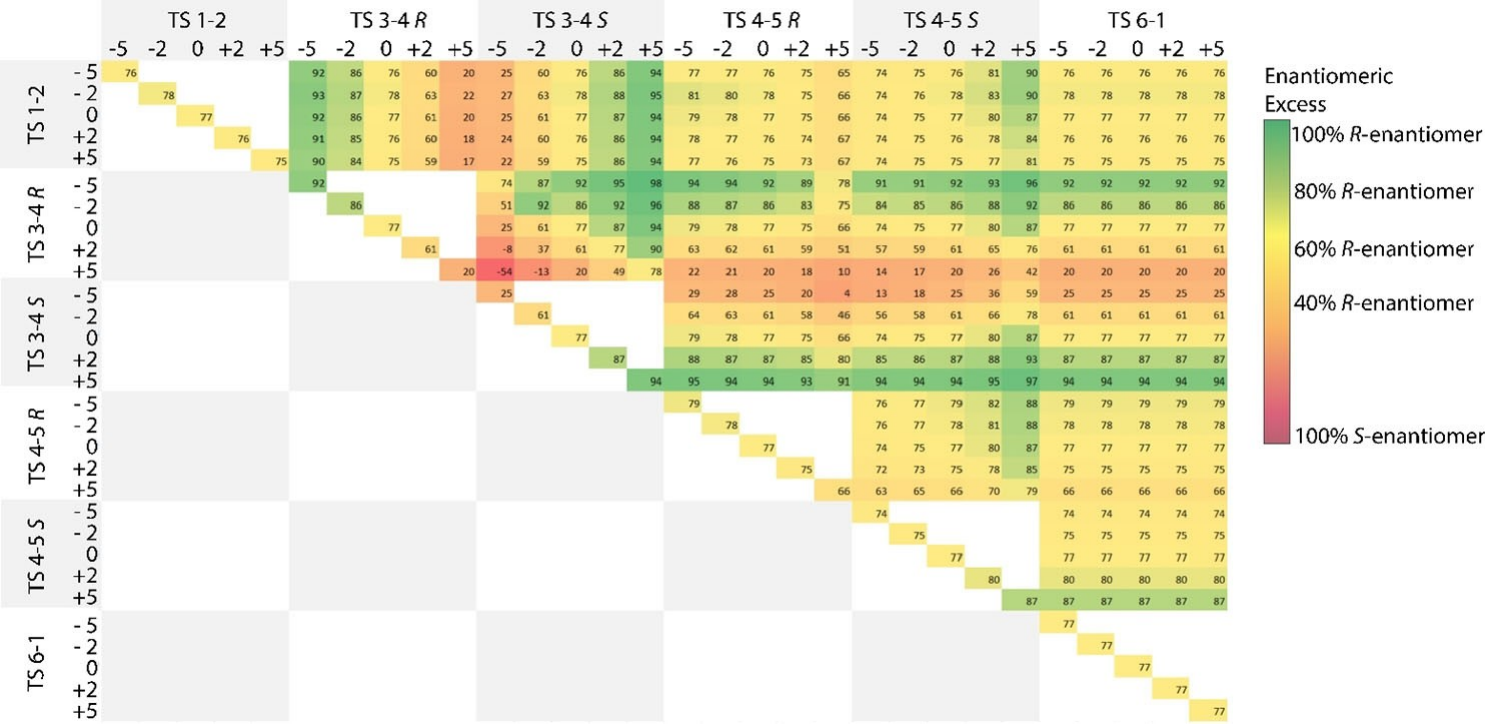
Multidimensional analysis of the transition state barriers (TS). Barriers were varied by −5 to 5 kJ mol^−1^. Enantiomeric excess is displayed by a color scale, dark green indicating high selectivity for the R‐enantiomer and red indicating a dominance of the S‐alcohol.

Next, we evaluated the sensitivity of the MKM‐predicted e.e. to the variations in the computed energetics of the individual steps in the reaction mechanism. Figure 4 shows how the predicted e.e. change upon variations of the computed free energy barriers for different elementary steps with increments in the range from −5 to +5 kJ mol^−1^. A maximum of two transition states were varied at the same instance in this analysis that are shown on the x‐ and y‐ axis of the diagram. The diagonal of Figure 4 therefore shows variations for the heights of the barriers of the individual elementary steps.

The most noticeable changes of the enantiomeric excess upon variation of one transition state barrier is observed for the enantiodetermining step (**TS 3**–**4**). An increase or decrease of this barrier by only 2 kJ mol^−1^ leads to e.e.’s of 61 % to 87 % (Figure 4). When the variation in the barrier height is increased to 5 kJ mol^−1^ (i. e., “the chemical accuracy”) the resulting e.e. range covers 20 % to 94 % as expected from the eq. 3 (Figure 1). The variations in the barrier heights for the other steps in the catalytic mechanism also affect the predicted e.e. but to a lesser extent. Alterations in the two transition states that are non‐stereospecific (**TS 1**–**2** and **TS 6**–**1**) have little to no effect on the predicted enantioselectivity. On the other hand, the variations in the alkoxide‐elimination step (**TS 4**–**5**) affect notably the predicted e.e. If the production of R‐phenylethanol (**TS 4**–**5R**: −5 kJ mol^−1^) is made more favorable the e.e. increases (<87 %), and a significant lower e.e. is achieved when the formation of the S‐enantiomer (**TS 4**–**5S**: −5 kJ mol^−1^) is enhanced (<67 %).

The simultaneous change of two barriers gives rise to even more significant variations in the predicted enantioselectivity. Again, the two reaction steps involved in the formation of the isopropoxide resting state (**TS 6**–**1**) and Mn‐hydride catalytic species (**TS 1**–**2**), do not affect the enantioselectivity.

**Figure 5:**
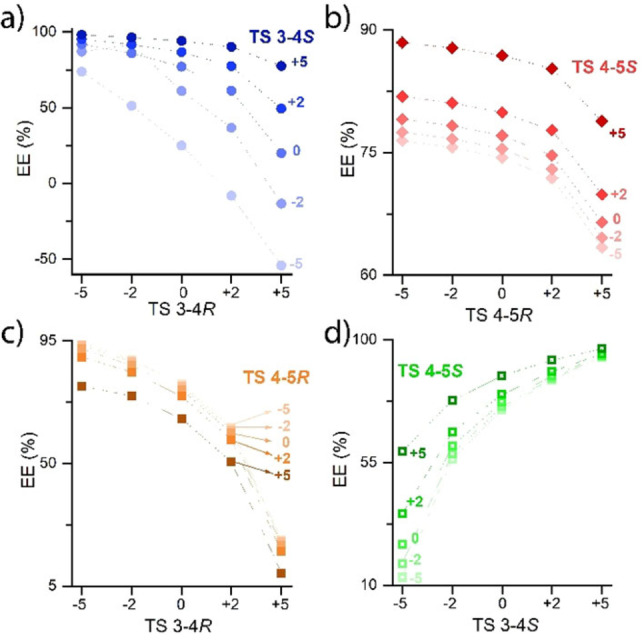
The effect of variation of two transition state barriers on the enantiomeric excess. The x‐axis illustrates the changes from −5 to +5 kJ mol^−1^ in the first TS barrier. The changes in the second TS barrier are plotted in five different scattering plots, also varying from −5 to +5 kJ mol^−1^, showing the resulting e.e. on the y‐axis.

Especially interesting are comparisons of simultaneous variations of barriers in the R‐ and S‐pathway or simultaneous variations in the enantiodetermining and alkoxide‐elimination step. This is illustrated in more detail in Figure 5, where we visualize the enantiomeric excess achieved upon variation of two transition states. Considering the enantiodetermining step (**TS 3**–**4**) a large range in the values of enantiomeric excess is observed (Figure 5a).

The highest e.e. value (>98 %) is observed when the barrier for the formation of R‐phenylethanol is decreased while the barrier for S‐phenylethanol is increased (**TS 3**–**4R**: −5 kJ mol^−1^ & **TS 3**–**4S**: +5 kJ mol^−1^), enhancing the ΔΔG of the two enantiomers by 10 kJ mol^−1^. The lowest and inverse e.e. (<−54) is detected in the other extreme case, in which the formation of the S‐enantiomer is made more favorable while simultaneously lowering the barrier of the R‐path (**TS 3**–**4R**: +5 kJ mol^−1^ & **TS 3**–**4S**: −5 kJ mol^−1^). Small variations in the barrier height of the alkoxide‐elimination step (**TS 4**–**5**, Figure 5b) lead to a range in e.e. of 63 % to 88 %. This range of e.e. is narrower than that observed upon the variation of the enantiodetermining step only.

Regarding simultaneous variations of alkoxide‐elimination and enantiodetermining steps, we can see that the alkoxide‐elimination step can add to the effect that was already observed during the increase and decrease of the barrier of the enantiodetermining step (Figure 5c and 5d). The lowest e.e. value was observed when only the barrier of the enantiodetermining step (**TS 3**–**4R**: +5 kJ mol^−1^) was changed by 20 %. When the alkoxide‐elimination step is altered concurrently (**TS 3**–**4S**: −5 kJ mol^−1^ & **TS 4**–**5R**: +5 kJ mol^−1^) the selectivity can be decreased further to 4 % e.e.

The analysis of the enantioselective excess shows that uncertainties in the computed free energy barriers for the stereo‐inducing steps have a very significant effect on the selectivity. Especially the enantiodetermining reaction step dictates the selectivity of the reaction and is therefore most susceptible to changes in the barrier. Furthermore, our analysis demonstrates that the energetics of the alkoxide‐elimination step also has a significant effect, introducing a change in selectivity by ca. 10 % when altered on its own and an additional change of 15 % e.e. when the respective barriers are varied simultaneously with those of the enantiodetermining step.

The kinetics of the alkoxide‐elimination step has been investigated in more detail. Figure S9 shows the reaction rates of the step for the forward and backward reactions as function of conversion. Interestingly, the forward reaction of the alkoxide‐elimination step proceeding through the *R*‐intermediates is a factor 15 faster than the backward reaction. The rate increases initially and when higher conversions are achieved decreases. The significant difference between the forward and backward reaction rate indicates why the alkoxide‐elimination step contributes to changes in enantiomeric excess. Alteration of the reaction barrier contributes significantly more to the forward reaction rate than the backward rate.

**Figure 6:**
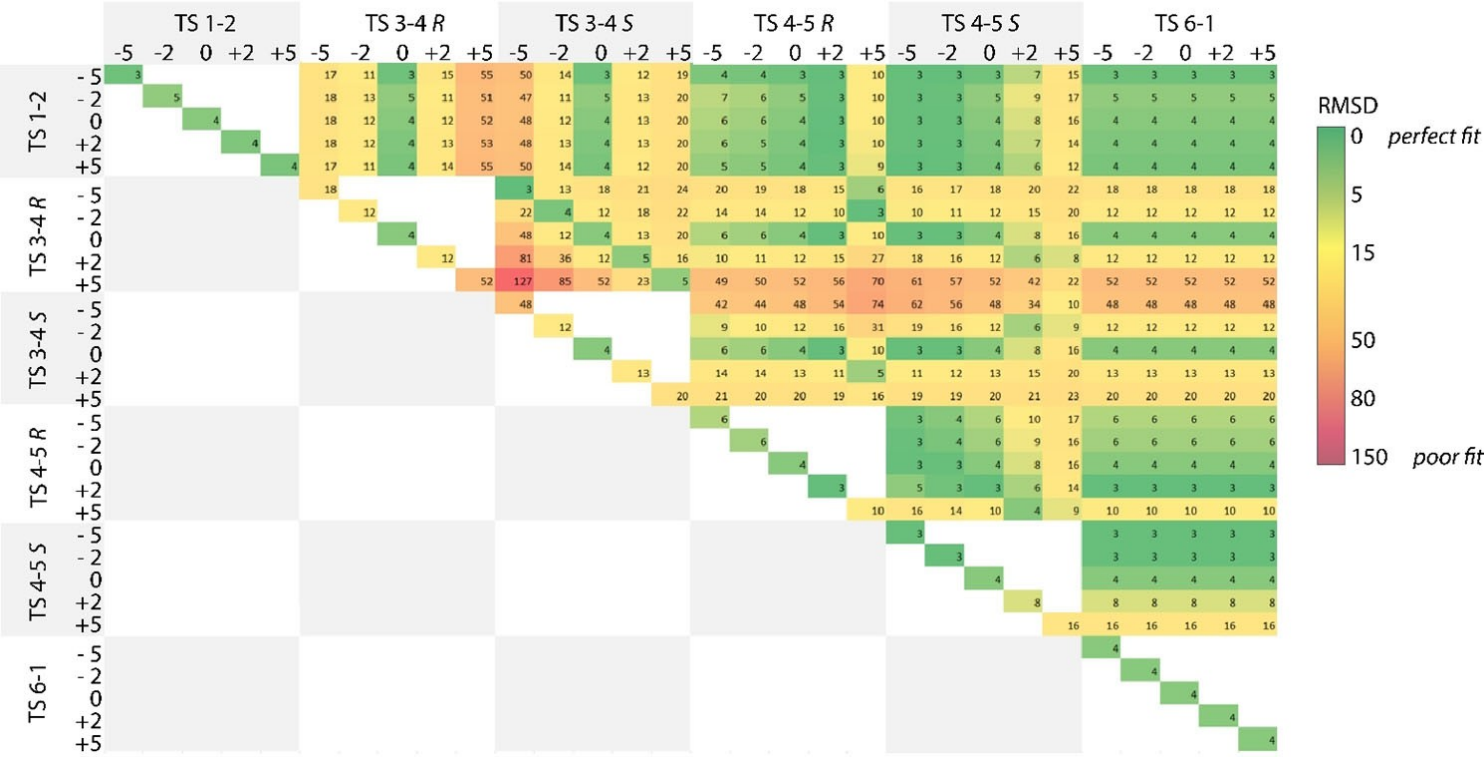
Root mean square deviation analysis of the e.e. versus conversion trajectory with variations in transition state barriers. Barriers were varied by −5 to 5 kJ mol^−1^. The RMSD is displayed by a color scale, dark green indicating a good fit and red representing a poor fit with the experimental data.

Next, the evolution of enantiomeric excess with substrate conversions was compared to data from the kinetic experiment. To analyze the data comprehensibly, the experimental e.e. at different conversion was compared to the data points obtained in the microkinetic model. The root mean square difference (RMSD) was calculated for these points and an analysis of changes in the trajectory was conducted when changing the barrier of transition states by 5, 2, −2 and −5 kJ mol^−1^ as shown in Figure 6. An example of how the MKM trajectory evolves for the original computed barriers and the best fit determined when changing the transition state energies compared to the experimentally determined data points is illustrated in the Supporting Information. The analysis of the changes in RMSD values is displayed in Figure 6. The alteration of computed transition states by 5 kJ mol^−1^ in one transition state can change the trajectory from a good fit shown in green to a poor fit depicted in red. For the original unaltered pathway, an RMSD of 4.4 is observed.

Results of alterations in a single transition state are shown on the diagonal axis. The greatest effect is again observed by the transition state introducing stereoselectivity (**TS 3**–**4**), inducing an RMSD value of almost 52 upon the increase of the respective **TS 3**–**4R** barrier by 5 kJ mol^−1^. Also, the alkoxide‐elimination step (**TS 4**–**5)** has a significant effect on the trajectory, changing the RMSD to almost 16. In line with the prior analysis, the final step in the cycle **TS 6**–**1** has no effect on the predictive power of the MKM. However, when the first reaction step (**TS 1**–**2**), in which the Mn‐hydride is formed, is reduced by 5 kJ mol^−1^ it results in the best fit (RMSD=3.2). By reducing the barrier, an initially higher e.e. value is achieved, which is also observed in the experimental data array. At 15 % ketone conversion the e.e. value accounts to 85 %, which decreases in time until it levels off at around 77 %.

The RMSD analysis of the variation of two concurrent reaction steps is in line with the results observed in the enantiomeric excess analysis. The stereospecific transition states (**TS 3‐4**, **TS 4**–**5**) have the greatest impact on the trajectory, while varying the two other barriers (**TS 1**–**2**, **TS 6**–**1**) leads to little deviation from the trajectory. Conversely, when altering the reaction step leading to the formation of R‐ and S‐phenylethanol simultaneously, the trajectory can be influenced significantly. Looking at the enantiodetermining step (**TS 3**–**4**), decreasing both enantiomers by 5 kJ mol^−1^ leads to a good fit with a RMSD with 2.6 (**TS 3**–**4R**: −5 kJ mol^−1^ & **TS 3**–**4S**: −5 kJ mol^−1^). An increase in both barriers of the rate determining step (**TS 4**–**5**) by 5 kJ mol^−1^ leads to a poorer fit with an RMSD of 6 (**TS 4**–**5R**: +5 kJ mol^−1^ & **TS 4**–**5S**: +5 kJ mol^−1^). Importantly, these changes do not affect the ΔΔG^≠^ differences for the competing reaction channels. Often higher enantioselectivities are achieved at lower conversions. The RMSD analysis shows how well the theoretical model mimics the experimentally determined trajectory.

## Conclusion

Herein we analyzed the impact of the uncertainties in computed barriers and detailization of the kinetic model on the predicted enantioselectivity of a model ketone transfer hydrogenation reaction by a homogeneous Mn(I)‐ diamine catalyst. During the analysis we have focused on comparing the enantiomeric excess achieved during the progress of the reaction to concentration profiles from the analysis from microkinetic modeling. The predicted trajectory and the achieved enantiomeric excess at different stages of the reaction was compared to experimental data.

The microkinetic model that was built upon the reaction energy diagram calculate by DFT confirms that the enantiodetermining step influences the selectivity to the greatest extent. It is also important that the variation in the barrier heights without changing the absolute ΔΔG^≠^ can lead to an enhanced enantiomeric excess: when changing **TS 3**–**4R** and **TS 3**–**4S** simultaneously the enantiomeric excess varies between 74 to 92 %. The alkoxide‐elimination step can also vary the enantioselective excess significantly. The final e.e. is least influence by the two non‐stereospecific steps.

However, the RMSD analysis showed that the time evolution of the enantiomeric excess can be affected by steps which are not affecting the final selectivity. This is the case since the enantioselective excess varies throughout the reaction trajectory. Also here, the trajectory is most significantly influenced by the enantiodetermining step, followed by the alkoxide‐elimination step.

In conclusion, we showed that the variations of the free energy barriers within the DFT uncertainty range of 5 kJ mol^−1^ can give rise to a complete reverse of the predicted enantioselectivity. Therefore, our analysis shows that an uncertainty of 5 kJ mol^−1^ in one transition state makes it impossible to predict reliably the enantioselective excess.

## Experimental Section

All DFT calculations were carried out with the Gaussian16 C0.1 package.^[56]^ The geometries of reaction intermediates and transition states were optimized with the PBE0‐D3(BJ)/ def2‐TZVP functional and basis set with a SMD solvent correction for isopropanol.^[57]^ An ultrafine grid was uniformly used. The nature of each stationary point was confirmed by frequency analysis in which zero imaginary frequencies for minima and one for transition states were found. Reaction (ΔE) and activation energies (ΔE^≠^) reported in the Supporting Information were corrected for zero‐point energy (ZPE) from the normal‐mode frequency analysis The finite temperature, enthalpy and entropic corrections to Gibbs free energies were computed from the results of normal mode analysis at a temperature (T) of 333.15 K. All Mn(I) complexes were considered in the singlet spin state only. Free energies in solution were computed by the SMD method within the ideal solution model approximation and the standard state of 1 mol L^−1^. The condition‐ and concentration dependencies were explicitly accounted for in the kinetic expressions only.

For the microkinetic model, an in‐house python script was used, and the reaction kinetics were derived for a batch‐type reactor (see Section S1 of the Supporting Information). Reaction rate constants were calculated using the Eyring equation. Reaction conditions and initial concentrations were adapted from the experimental conditions. The enantiomeric excess was determined at 70 % conversion by the equation given in the previous section, using the concentrations of *R*‐ and *S*‐phenylethanol. To determine the root mean square deviation (RMSD) the points of conversion and the measured e.e. from experimental data were compared to the nearest point of conversion in the microkinetic trajectory. The data points were processed with the formula for RMSD:

$$RMSD=\;\sqrt{\frac{\sum_{n=1}^{N}{\left(\hat{y_{n}}-y_{n}\right)}^{2}}{N}}$$

where ($\hat{y_{n}}$ ) is the predicted enantiomeric excess from the microkinetic model, $y_{n}$ is the observed e.e. from the experimental trajectory and N is the number of total data points regarded, which was 9.

The exhaustive conformational transition state search was carried out using QM‐based molecular dynamics using the PBE functional in cp2k.^[58]^ The lowest 50 conformers were further investigated, and the two structures with the greatest conformational differences were optimized with DFT according to the description above.

A copy of the accompanying data is freely available at *4TU.ResearchData* repository via DOI: 10.4121/14637681.

## Conflict of interest

The authors declare no conflict of interest.

## Supporting information

As a service to our authors and readers, this journal provides supporting information supplied by the authors. Such materials are peer reviewed and may be re‐organized for online delivery, but are not copy‐edited or typeset. Technical support issues arising from supporting information (other than missing files) should be addressed to the authors.

Supporting Information
