# Supplementary material for: The Impact of Computational Uncertainties on the Enantioselectivity Predictions: A Microkinetic Modeling of Ketone Transfer Hydrogenation with a Noyori‐type Mn‐diamine Catalyst
Source: ChemCatChem. 2021 Jun 10;13(15):3517–24. doi: 10.1002/cctc.202100341 (PMC8453751; doi:10.1002/cctc.202100341)
Supplement: Supplementary file 1 — Supporting Information [file CCTC-13-3517-s001.pdf]

ChemCatChem

Supporting Information

The Impact of Computational Uncertainties on the Enantioselectivity Predictions: A Microkinetic Modeling of Ketone Transfer Hydrogenation with a Noyori-type Mn-diamine Catalyst

Annika M. Krieger* and Evgeny A. Pidko*

Table of Contents

S1. Gibbs Free Energies to Rate Constants 3
S2. Experimental trajectory 3
S3. Kinetic trajectories 3
S4. Elementray reactions 4
S5. Degree of rate control 8
S6. Comparison NH1 and NH2 channel 8
S7. Comparison Backward to Forward Reaction Contribution (R and S) 9
S8. Exhaustive conformational transition state search 13
S9. Energetics of optimized structures 16

S1. Gibbs Free Energies to Rate Constants

To determine rate constants in the microkinetic model, the Eyring equation is considered to calculate the rate constants:

$$k = \frac{k_B T}{h} e^{(-\frac{\Delta G}{RT})}$$

S2. Experimental trajectory

Below, the experimental trajectory of e.e. at different coversions is summarized (from Putten et al. *Organometallics* **2019**, *38*, 3187). Experiment was carried out at 333.15K, with 0.5mmol acetophenone, 0.5mol% catalyst, 3.8mL 2-propanol. These parameters were translated to the microkinetic model.

Conversion (%)	Enantioselective Excess (%)
1	72
4	74
7	75
13	82
20	74
39	74
54	74
67	74
85	73

S3. Kinetic trajectories

The kinetic trajectories of enantiomeric excess to conversion were compared to the experimental trajectory mentioned above to obtain the enantiomeric excess at 70% conversion and the RMSD between the data points. A few examples of the trajectories are illustrated below.

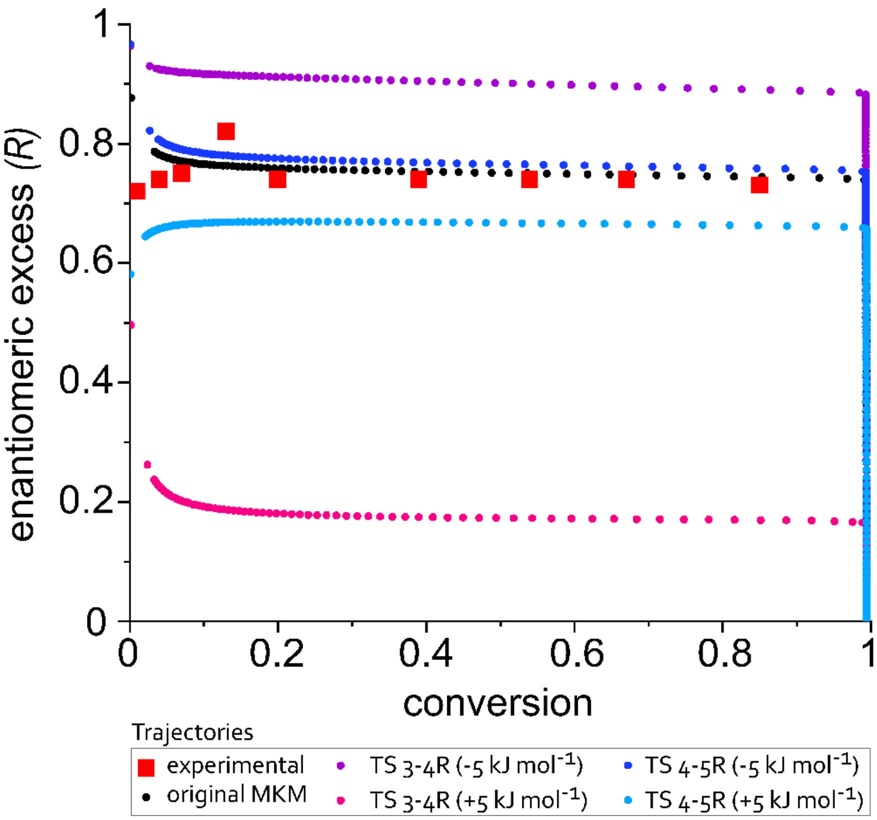

Figure S1. Trajectories of enantiomeric excess developing as a function of conversion of the experimental data (red) and various microkinetic model runs.

S4. Elementray reactions

Below, the reaction network and the corresponding elementary reactions for the ketone reduction pathway are summarized. The Gibbs free energies profile are presented for both the NH1 and NH2 channel.

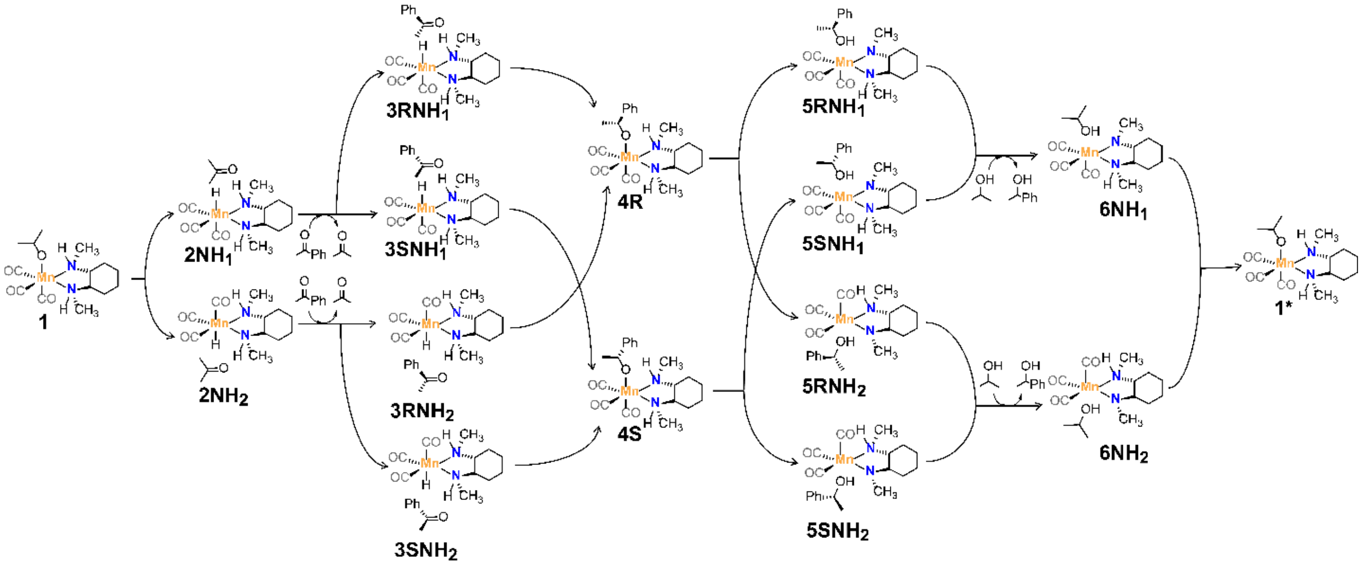

Figure S2: Elementary reaction steps

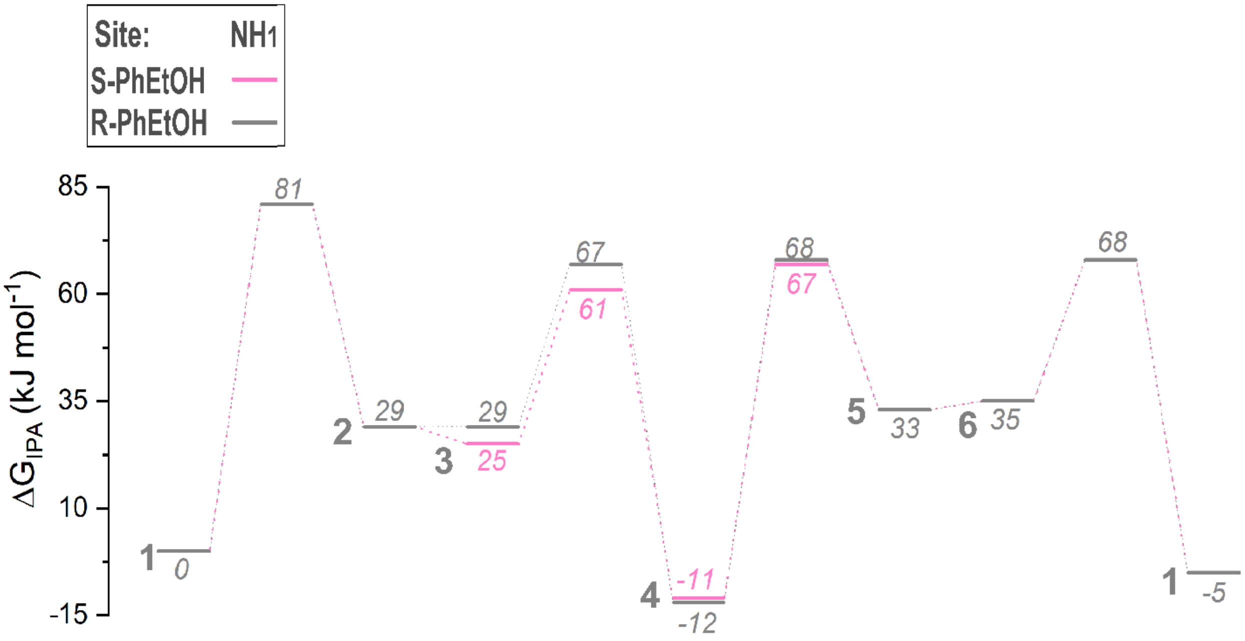

Figure S3: Free energy diagram of the reduction of acetophenone proceeding through NH1 channel

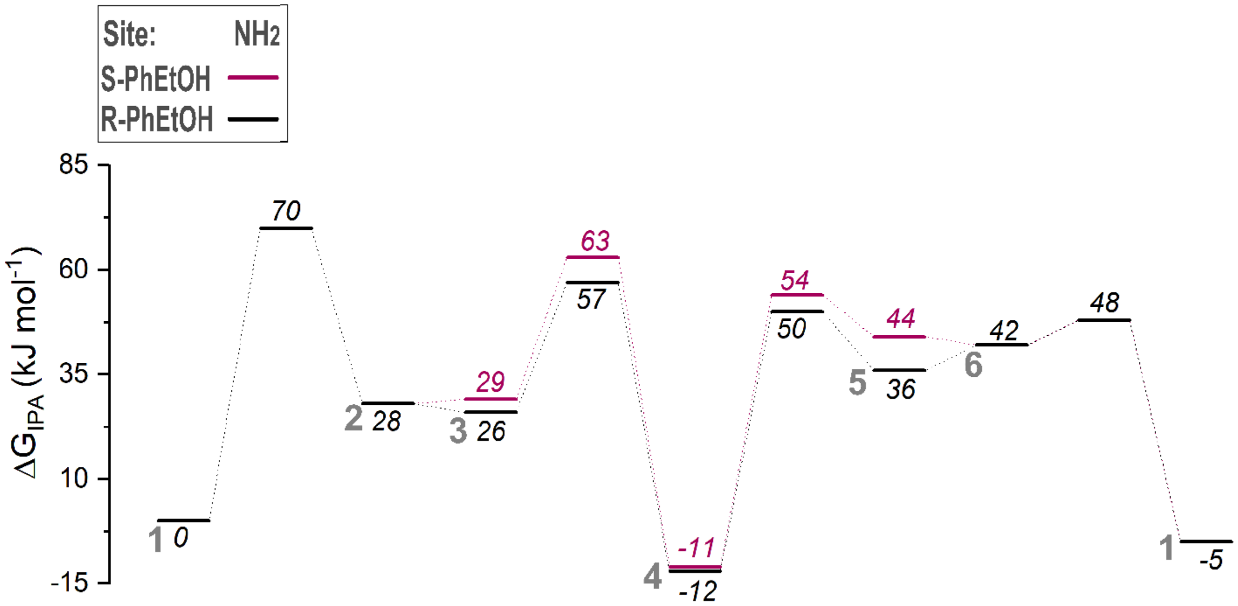

Figure S4: Free energy diagram of the reduction of acetophenone proceeding through NH2 channel

$$\frac{d[1]}{dt} == -(k_{1-\mathrm{2NH1}} * [1] - k_{\mathrm{2NH1}-1} * [\mathrm{2NH1}]) - (k_{1-\mathrm{2NH2}} * [1] - k_{\mathrm{2NH2}-1} * [\mathrm{2NH2}]) + (k_{1-\mathrm{6NH1}} * [\mathrm{6NH1}] - k_{1-\mathrm{6NH1}} * [1]) + (k_{1-\mathrm{6NH2}} * [\mathrm{6NH2}] - k_{1-\mathrm{6NH2}} * [1])$$

$$\frac{d[\mathrm{2NH1}]}{dt} = +(k_{1-\mathrm{2NH1}} * [1] - k_{\mathrm{2NH1}-1} * [\mathrm{2NH1}]) - (k_{\mathrm{2NH1}-3R\mathrm{NH1}} * [\mathrm{2NH1}] * [\mathrm{acetophenone}] - k_{3R\mathrm{NH1}-\mathrm{2NH1}} * [3R\mathrm{NH1}] * [\mathrm{acetone}]) - (k_{\mathrm{2NH1}-3S\mathrm{NH1}} * [\mathrm{2NH1}] * [\mathrm{acetophenone}] - k_{3S\mathrm{NH1}-\mathrm{2NH1}} * [3S\mathrm{NH1}] * [\mathrm{acetone}])$$

$$\frac{d[\mathrm{2NH2}]}{dt} = +(k_{1-\mathrm{2NH2}} * [1] - k_{\mathrm{2NH2}-1} * [\mathrm{2NH2}]) - (k_{\mathrm{2NH2}-\mathrm{3RNH2}} * [\mathrm{2NH2}] * [\mathrm{acetophenone}] - k_{3R\mathrm{NH2}-\mathrm{2NH2}} * [3R\mathrm{NH2}] * [\mathrm{acetone}]) - (k_{\mathrm{2NH2}-3S\mathrm{NH2}} * [\mathrm{2NH2}] * [\mathrm{acetophenone}] - k_{3S\mathrm{NH2}-\mathrm{2NH2}} * [3S\mathrm{NH2}] * [\mathrm{acetone}])$$

$$\frac{d[3R\mathrm{NH1}]}{dt} = + (k_{\mathrm{2NH1}-\mathrm{3RNH1}} * [\mathrm{2NH1}] * [\mathrm{acetophenone}] - k_{3R\mathrm{NH1}-\mathrm{2NH1}} * [3R\mathrm{NH1}] * [\mathrm{acetone}]) - (k_{3R\mathrm{NH1}-4R} * [3R\mathrm{NH1}] - k_{4R-3R\mathrm{NH1}} * [4R])$$

$$\frac{d[3S\mathrm{NH1}]}{dt} = + (k_{\mathrm{2NH1}-3S\mathrm{NH1}} * [\mathrm{2NH1}] * [\mathrm{acetophenone}] - k_{3S\mathrm{NH1}-\mathrm{2NH1}} * [3S\mathrm{NH1}] * [\mathrm{acetone}]) - (k_{3S\mathrm{NH1}-4S} * [3S\mathrm{NH1}] - k_{4S-3S\mathrm{NH1}} * [4S])$$

$$\frac{d[3R\mathrm{NH2}]}{dt} = + (k_{\mathrm{2NH2}-\mathrm{3RNH2}} * [\mathrm{2NH2}] * [\mathrm{acetophenone}] - k_{3R\mathrm{NH2}-\mathrm{2NH2}} * [3R\mathrm{NH2}] * [\mathrm{acetone}]) - (k_{3R\mathrm{NH2}-4R} * [3R\mathrm{NH2}] - k_{4R-3R\mathrm{NH2}} * [4R])$$

$$\frac{d[3S\mathrm{NH2}]}{dt} = +\ (k_{2\mathrm{NH2}-3S\mathrm{NH2}} * [\mathrm{2NH2}] * [\mathrm{acetophenone}] - k_{3S\mathrm{NH2}-2\mathrm{NH2}} * [3S\mathrm{NH2}] * [\mathrm{acetone}]) - (k_{3S\mathrm{NH2}-4S} * [3S\mathrm{NH2}] - k_{4S-3S\mathrm{NH2}} * [4S])$$

$$\frac{d[4R]}{dt} = +(k_{3R\mathrm{NH1}-4R} * [3R\mathrm{NH1}] - k_{4R-3R\mathrm{NH1}} * [4R]) + (k_{3R\mathrm{NH2}-4R} * [3R\mathrm{NH2}] - k_{4R-3R\mathrm{NH1}} * [4R]) - (k_{4R-5R\mathrm{NH1}} * [4R] - k_{5R\mathrm{NH1}-4R} * [5R\mathrm{NH1}]) - (k_{4R-5R\mathrm{NH2}} * [4R] - k_{5R\mathrm{NH2}-4R} * [5R\mathrm{NH2}])$$

$$\frac{d[4S]}{dt} = +(k_{3S\mathrm{NH1}-4S} * [3S\mathrm{NH1}] - k_{4S-3S\mathrm{NH1}} * [4S]) + (k_{3S\mathrm{NH2}-4S} * [3S\mathrm{NH2}] - k_{4S-3S\mathrm{NH1}} * [4S]) - (k_{4S-5S\mathrm{NH1}} * [4S] - k_{5S\mathrm{NH1}-4S} * [5S\mathrm{NH1}]) - (k_{4S-5S\mathrm{NH2}} * [4S] - k_{5S\mathrm{NH2}-4S} * [5S\mathrm{NH2}])$$

$$\frac{d[5R\mathrm{NH1}]}{dt} = +\ (k_{4R-5R\mathrm{NH1}} * [4R] - k_{5R\mathrm{NH1}-4R} * [5R\mathrm{NH1}]) - (k_{5R\mathrm{NH1}-6\mathrm{NH1}} * [5R\mathrm{NH1}] * [\mathrm{IPA}] - k_{6\mathrm{NH1}-5R\mathrm{NH1}} * [\mathrm{6NH1}] * [R\mathrm{PhCH(OH)CH3}])$$

$$\frac{d[5S\mathrm{NH1}]}{dt} = +\ (k_{4S-5S\mathrm{NH1}} * [4S] - k_{5S\mathrm{NH1}-4S} * [5S\mathrm{NH1}]) - (k_{5S\mathrm{NH1}-6\mathrm{NH1}} * [5S\mathrm{NH1}] * [\mathrm{IPA}] - k_{6\mathrm{NH1}-5S\mathrm{NH1}} * [\mathrm{6NH1}] * [S\mathrm{PhCH(OH)CH3}])$$

$$\frac{d[5R\mathrm{NH2}]}{dt} = +\ (k_{4R-5R\mathrm{NH2}} * [4R] - k_{5R\mathrm{NH2}-4R} * [5R\mathrm{NH2}]) - (k_{5R\mathrm{NH2}-6\mathrm{NH2}} * [5R\mathrm{NH2}] * [\mathrm{IPA}] - k_{6\mathrm{NH2}-5R\mathrm{NH2}} * [\mathrm{6NH2}] * [R\mathrm{PhCH(OH)CH3}])$$

$$\frac{d[5S\mathrm{NH2}]}{dt} = +\ (k_{4S-5S\mathrm{NH2}} * [4S] - k_{5S\mathrm{NH2}-4S} * [5S\mathrm{NH2}]) - (k_{5S\mathrm{NH2}-6\mathrm{NH2}} * [5S\mathrm{NH2}] * [\mathrm{IPA}] - k_{6\mathrm{NH2}-5S\mathrm{NH2}} * [\mathrm{6NH2}] * [S\mathrm{PhCH(OH)CH3}])$$

$$\frac{d[\mathrm{6NH1}]}{dt} = +\ (k_{5R\mathrm{NH1}-6\mathrm{NH1}} * [5R\mathrm{NH1}] * [\mathrm{IPA}] - k_{6\mathrm{NH1}-5R\mathrm{NH1}} * [\mathrm{6NH1}] * [R\mathrm{PhCH(OH)CH3}]) + (k_{5S\mathrm{NH1}-6\mathrm{NH1}} * [5S\mathrm{NH1}] * [\mathrm{IPA}] - k_{6\mathrm{NH1}-5S\mathrm{NH1}} * [\mathrm{6NH1}] * [S\mathrm{PhCH(OH)CH3}]) - (k_{6\mathrm{NH1}-1} * [\mathrm{6NH1}] - k_{1-6\mathrm{NH1}} * [1]])$$

$$\frac{d[\mathrm{6NH2}]}{dt} = +\ (k_{5R\mathrm{NH2}-6\mathrm{NH2}} * [5R\mathrm{NH2}] * [\mathrm{IPA}] - k_{6\mathrm{NH2}-5R\mathrm{NH2}} * [\mathrm{6NH2}] * [R\mathrm{PhCH(OH)CH3}]) + (k_{5S\mathrm{NH2}-6\mathrm{NH2}} * [5S\mathrm{NH2}] * [\mathrm{IPA}] - k_{6\mathrm{NH2}-5S\mathrm{NH2}} * [\mathrm{6NH2}] * [S\mathrm{PhCH(OH)CH3}]) - (k_{6\mathrm{NH2}-1} * [\mathrm{6NH2}] - k_{1-6\mathrm{NH2}} * [1]])$$

$$\frac{d[\mathrm{acetophenone}]}{dt} = -(k_{2\mathrm{NH1}-3R\mathrm{NH1}} * [\mathrm{2NH1}] * [\mathrm{acetophenone}] - k_{3R\mathrm{NH1}-2\mathrm{NH1}} * [3R\mathrm{NH1}] * [\mathrm{acetone}]) - (k_{2\mathrm{NH1}-3S\mathrm{NH1}} * [\mathrm{2NH1}] * [\mathrm{acetophenone}] - k_{3S\mathrm{NH1}-2\mathrm{NH1}} * [3S\mathrm{NH1}] * [\mathrm{acetone}]) - (k_{2\mathrm{NH2}-3R\mathrm{NH2}} * [\mathrm{2NH2}] * [\mathrm{acetophenone}] - k_{3R\mathrm{NH2}-2\mathrm{NH2}} * [3R\mathrm{NH2}] * [\mathrm{acetone}]) - (k_{2\mathrm{NH2}-3S\mathrm{NH2}} * [\mathrm{2NH2}] * [\mathrm{acetophenone}] - k_{3S\mathrm{NH2}-2\mathrm{NH2}} * [3S\mathrm{NH2}] * [\mathrm{acetone}])$$

$$\frac{d[acetone]}{dt} = +(k_{2\mathrm{NH1}-3R\mathrm{NH1}} * [\mathrm{2NH1}] * [\mathrm{acetophenone}] - k_{3R\mathrm{NH1}-2\mathrm{NH1}} * [3R\mathrm{NH1}] * [\mathrm{acetone}]) + (k_{2\mathrm{NH1}-3S\mathrm{NH1}} * [\mathrm{2NH1}] * [\mathrm{acetophenone}] - k_{3S\mathrm{NH1}-2\mathrm{NH1}} * [3S\mathrm{NH1}] * [\mathrm{acetone}]) + (k_{2\mathrm{NH2}-3R\mathrm{NH2}} * [\mathrm{2NH2}] * [\mathrm{acetophenone}] - k_{3R\mathrm{NH2}-2\mathrm{NH2}} * [3R\mathrm{NH2}] * [\mathrm{acetone}]) + (k_{2\mathrm{NH2}-3S\mathrm{NH2}} * [\mathrm{2NH2}] * [\mathrm{acetophenone}] - k_{3S\mathrm{NH2}-2\mathrm{NH2}} * [3S\mathrm{NH2}] * [\mathrm{acetone}])$$

$$\frac{d[IPA]}{dt} = -(k_{5R\text{NH1}-6\text{NH1}} * [5R\text{NH1}] * [\text{IPA}] - k_{6\text{NH1}-5R\text{NH1}} * [6\text{NH1}] * [R\text{PhCH(OH)CH3}])$$
$$- (k_{5S\text{NH1}-6\text{NH1}} * [5S\text{NH1}] * [\text{IPA}] - k_{6\text{NH1}-5S\text{NH1}} * [6\text{NH1}] * [S\text{PhCH(OH)CH3}])$$
$$- (\text{k}_{5R\text{NH2}-6\text{NH2}} * [5R\text{NH2}] * [\text{IPA}] - k_{6\text{NH2}-5R\text{NH2}} * [6\text{NH2}] * [R\text{PhCH(OH)CH3}])$$
$$- (k_{5S\text{NH2}-6\text{NH2}} * [5S\text{NH2}] * [\text{IPA}] - k_{6\text{NH2}-5S\text{NH2}} * [6\text{NH2}] * [S\text{PhCH(OH)CH3}])$$

$$\frac{d[R\text{PhCH(OH)CH3}]}{dt}$$
$$= +(k_{5R\text{NH1}-6\text{NH1}} * [5R\text{NH1}] * [\text{IPA}] - k_{6\text{NH1}-5R\text{NH1}} * [6\text{NH1}] * [R\text{PhCH(OH)CH3}])$$
$$+ (k_{5R\text{NH2}-6\text{NH2}} * [5R\text{NH2}] * [\text{IPA}] - k_{6\text{NH2}-5R\text{NH2}} * [6\text{NH2}] * [R\text{PhCH(OH)CH3}])$$

$$\frac{d[S\text{PhCH(OH)CH3}]}{dt}$$
$$= +(k_{5S\text{NH1}-6\text{NH1}} * [5S\text{NH1}] * [\text{IPA}] - k_{6\text{NH1}-5S\text{NH1}} * [6\text{NH1}] * [S\text{PhCH(OH)CH3}])$$
$$+ (k_{5S\text{NH2}-6\text{NH2}} * [5S\text{NH2}] * [\text{IPA}] - k_{6\text{NH2}-5S\text{NH2}} * [6\text{NH2}] * [S\text{PhCH(OH)CH3}])$$

S5. Degree of rate control

The degree of rate control grouped in the general reaction steps is summarized in the table below:

Reaction step	DRC
TS 1-2	0.88
TS 3-4	0
TS 4-5	0.12
TS 6-1	0

S6. Comparison NH1 and NH2 channel

The proposed reaction networks implies that the reaction can follow several channels. We compared the rate of the reaction proceeding solely through the NH1 and the NH2 channel. Our analysis reveals that the reduction reaction through the NH2 channel proceeds much faster than through the NH1 channel With the NH1 to NH2 ratio of approximately 0.01. The enantiomeric excess at 70% conversion is 41% S-enantiomer when proceeding through the NH1-channel and 80% R-enantiomer when proceeding through the NH2-channel. The reaction proceeding through the NH1 channel can therefore contribute to the deterioration of the enantiomeric excess of R-phenyl ethanol.

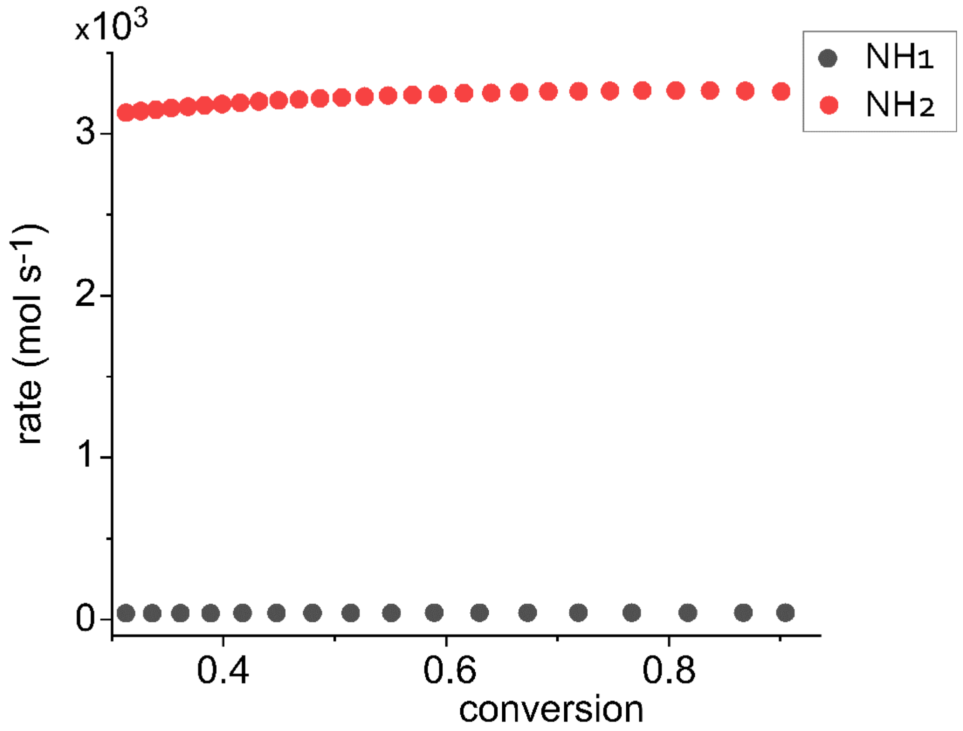

Figure S5. Rate of phenylethanol production as a function of conversion proceeding separately through NH1 channel (black) or NH2 channel (red).

S7. Comparison Backward to Forward Reaction Contribution (R and S)

Here reaction rates for the formation of R-phenylethanol and S-phenylethanol are compared. The illustrations below account for the reaction rates leading to the formation of R- and S-enantiomer as a function of conversion. The change of the netto reaction reate for the R-enantiomer is displayed to account for the changes that occur as the reaction proceeds.

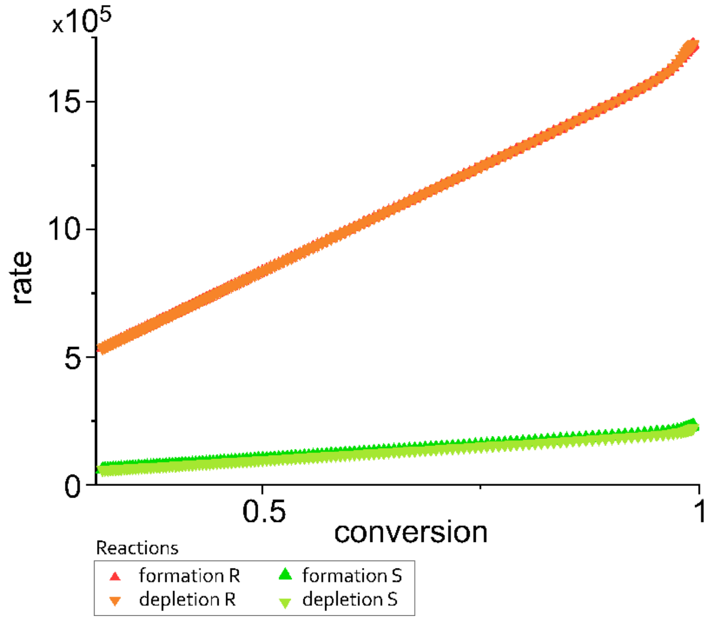

Figure S6. Reaction rates of the formation of R- and S-phenylethanol and the consumption of R- and S-phenylethanol as a function of conversion

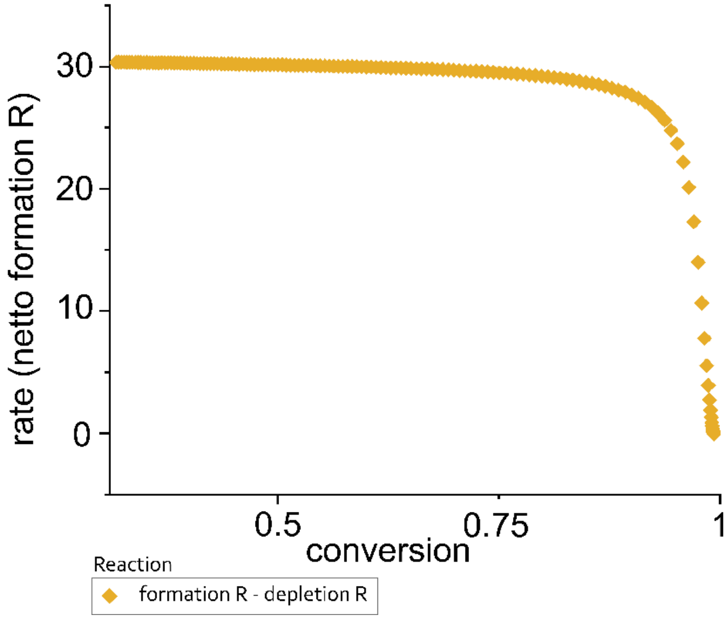

Figure S7. Netto reaction rate of the formation of R-phenylethanol minus its consumption as a function of conversion

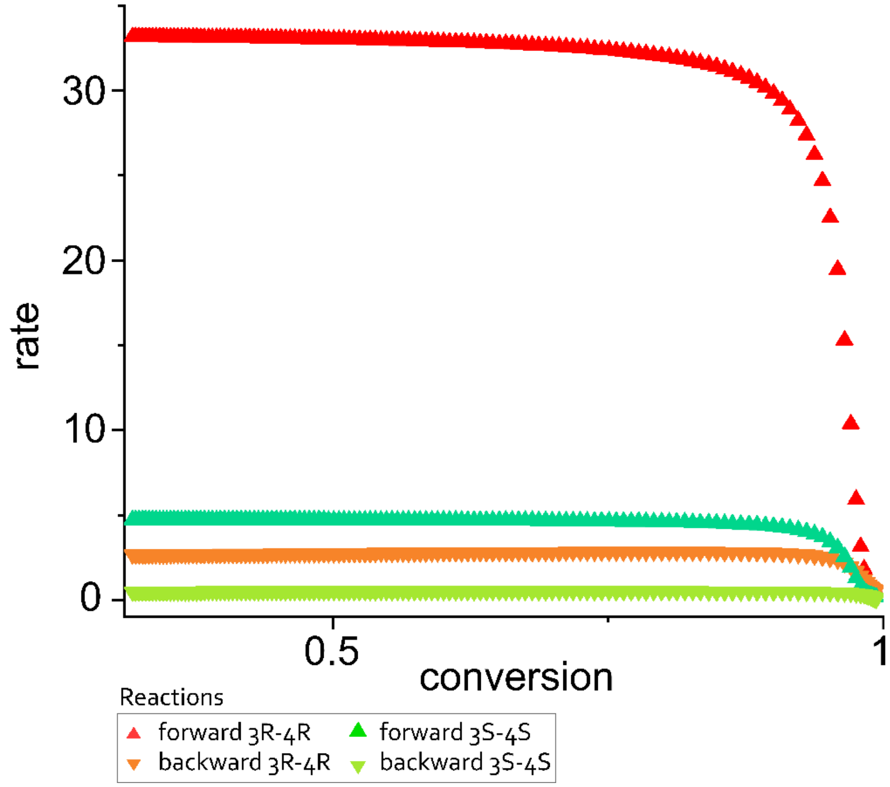

Figure S8. Reaction rates of the forward and backward reaction of the conversion of 3 to 4 as a function of conversion

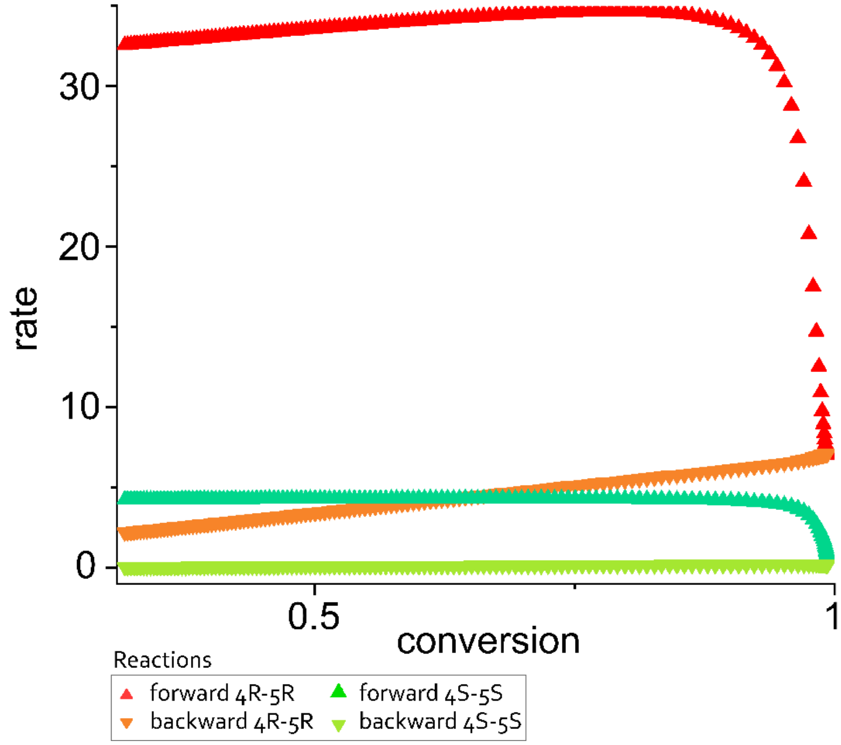

Figure S9. Reaction rates of the forward and backward reaction of the conversion of 4 to 5 as a function of conversion

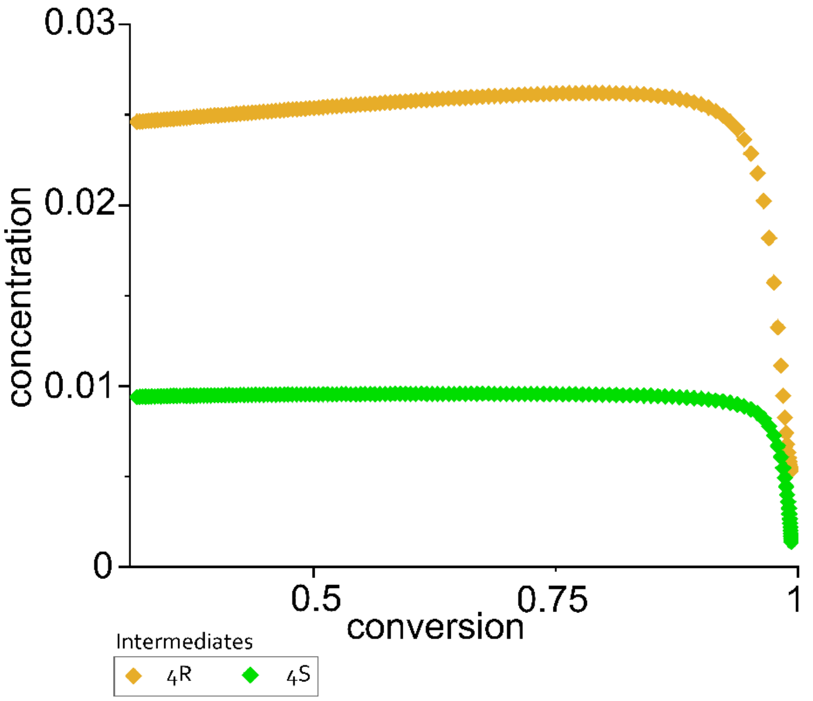

Figure S10. Concentration profile of intermediates 4R and 4S as a function of conversion

Below, we compare the enantiomeric excess at 70% conversion of the final product to the ratio of 4R to 4S intermediate (also reported as enantiomeric excess) when the TS 4-5 is varied:

Trajectroy	Enantioselective Excess (%)	Enantioselective Excess Int 4 (%)
Original	77	46
R-barrier: -5 kJ mol^{-1}	79	-10
R-barrier: -2 kJ mol^{-1}	78	23
R-barrier: +2 kJ mol^{-1}	75	66
R-barrier: +5 kJ mol^{-1}	66	83
S-barrier: -5 kJ mol^{-1}	74	86
S-barrier: -2 kJ mol^{-1}	75	68
S-barrier: +2 kJ mol^{-1}	79	17
S-barrier: +5 kJ mol^{-1}	86	-23
R- & S-barrier: -5 kJ mol^{-1}	76	60
R- & S-barrier: -2 kJ mol^{-1}	77	51
R- & S-barrier: +2 kJ mol^{-1}	78	43
R- & S-barrier: +5 kJ mol^{-1}	79	39

S8. Exhaustive conformational transition state search

For the search of new transition states (TS4-5R NH2) within the ensemble the following steps were followed.

1. A cubic solvent box (20Å x 20Å x 20Å) with isopropanol molecules surrounding the transition state structure was build using GROMACS. [1,2]
2. The bonding length between the substarte and catalyst were constrained according to the bonding parameters of the existing transition state. Mn-O at 2.60Å, O-H at 0.997Å, H-N at 1.75Å.
3. An ab initio MD run at 333.15K at NVT ensemble was carried out for 5000 steps in cp2k 6.1 software[3] with the quickstep module and orbital transformation for faster convergence. in the initial 500 steps velocity softerning was applied. The DZV-GTH-PADE basis set was applied to Mn, TZVP-GTH to all other atoms. All calculations were spin-polarized and PBE-D3(BJ) [4] correction was applied.
4. The 50 lowest conformers obtained in step 500 to 3000 were taken from the MD trajectory, optimized with constraints in DFT, and the structure resulting in the highest RMSD from the initial transition state structure was fully optimized in accordance to the other reported TS structures.

This search resulted in a 5 kJ mol^{-1} difference between the initial and the transition state found from the MD run. Energetics and xyz coordinates are reported below.

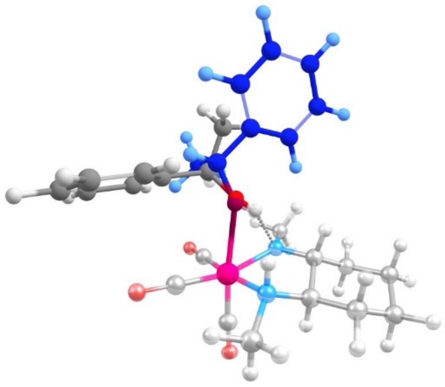

Grey structure				Blue structure			
Mn	-0.20587200	-0.81041000	0.47045700	Mn	-1.35932900	-1.14134600	0.08083200
O	-1.47520800	-3.32604900	1.12113800	O	-4.17880600	-1.08966100	0.72763300
O	1.31489600	-0.97730000	2.96652800	O	-0.97001000	-3.13021200	2.19272600
N	-1.27155600	-0.52665900	-1.28830600	N	-1.49944800	0.34000400	-1.36588300
C	0.71348700	-0.88395900	1.99020000	C	-1.09551200	-2.33774300	1.36874500
C	1.16508100	-1.60097400	-0.37101100	C	-1.50429700	-2.46929600	-1.10860300
O	2.05149000	-2.12504700	-0.88458800	O	-1.55585100	-3.34234000	-1.85731700
C	-0.93737200	-2.34193900	0.84717300	C	-3.05998700	-1.13965000	0.44862000
N	-1.40285800	0.57397100	1.07082400	N	-0.58588500	0.33024900	1.07820500
C	-2.52900600	0.90232700	0.19589300	C	-0.74438100	1.66836200	0.50660400
C	-2.64241400	-0.18610200	-0.86006300	C	-1.75840300	1.58998300	-0.62548400
H	-3.03023100	-1.08812900	-0.36768900	H	-2.75235500	1.46590600	-0.17449100
O	0.67173300	1.52550700	-0.36668300	O	1.00516200	-0.80092300	-0.70845200
C	1.83202300	1.91930900	0.36412700	C	2.25472800	-1.45011400	-0.52849500
H	1.69256300	1.66487200	1.42202300	H	2.43057700	-2.01601000	-1.44856800
C	2.04336200	3.41876100	0.24284500	C	2.22000800	-2.41923000	0.64375800
H	1.17643400	3.95450500	0.64172500	H	1.45008300	-3.17978100	0.48743900
H	2.93101400	3.73358400	0.80012800	H	3.18504100	-2.92269200	0.75213000
H	2.17553700	3.70223500	-0.80598200	H	2.00824300	-1.88634900	1.57677300
H	-2.31280300	1.85301800	-0.33322600	H	0.21924500	1.98875600	0.06085900
C	-3.60988300	0.21768700	-1.96309200	C	-1.75808500	2.87085500	-1.44735000
H	-3.23871200	1.12399300	-2.45970200	H	-0.76581000	3.01624800	-1.89453000
H	-3.68396400	-0.56464800	-2.72325200	H	-2.48034300	2.81143600	-2.26612200
C	-3.90007200	1.07922700	0.84848500	C	-1.17100200	2.79520600	1.44761400
H	-3.86192700	1.84790600	1.62469300	H	-0.47814300	2.87927300	2.28890400
H	-4.19347700	0.13977900	1.33604200	H	-2.16123100	2.56675200	1.86414700
C	-4.98815700	0.47018800	-1.35466600	C	-2.11222800	4.04842700	-0.54093900
C	-4.93099700	1.47208500	-0.20750700	C	-1.20977200	4.11827300	0.68563100
H	-4.66821500	2.46140500	-0.60530500	H	-0.18925300	4.37194200	0.36888800
H	-5.91925900	1.57285700	0.25363700	H	-1.54037200	4.92372900	1.35008400
H	-5.68039900	0.81690100	-2.12890600	H	-2.05902300	4.98451700	-1.10646700
H	-5.38578500	-0.48265800	-0.98105400	H	-3.15464700	3.93728000	-0.21440200
H	-0.85700000	0.30565700	-1.70966900	H	-0.55481000	0.38858300	-1.75126500
C	-1.22199500	-1.60219000	-2.28578800	C	-2.43074600	0.11425900	-2.47650200
H	-1.76304900	-1.34074100	-3.19902300	H	-2.38303500	0.91287700	-3.22150200
H	-0.18397800	-1.80511600	-2.54992900	H	-2.18698600	-0.82733800	-2.96916900
H	-1.65869800	-2.50923000	-1.86183300	H	-3.45003700	0.05134600	-2.08865300
H	-0.09868700	1.46053300	0.26037600	H	0.71926300	-0.31114200	0.11825300
C	-1.50651000	1.14678400	2.39720800	C	-0.18558000	0.32568100	2.46937200
H	-1.68074500	2.23390400	2.36250700	H	0.09166200	-0.68440600	2.77992800
H	-2.31769600	0.71264500	3.00147800	H	0.69477900	0.96240500	2.64801600
H	-0.57742600	0.99441600	2.95033900	H	-0.97336600	0.66998700	3.15743800
C	3.00984500	1.13068800	-0.14982000	C	3.37766000	-0.44694000	-0.37504700
C	3.84969500	0.45064300	0.72838300	C	4.62671700	-0.70540800	-0.93748100
C	3.26095700	1.05605900	-1.52081300	C	3.20043200	0.72720500	0.35702500
C	4.92411100	-0.29254900	0.24882500	C	5.67980600	0.18764600	-0.77135500
H	3.65072100	0.49005500	1.79560500	H	4.77343100	-1.61435300	-1.51501800
C	4.33049200	0.31283300	-2.00214400	C	4.25058600	1.62299900	0.52315200
H	2.60217800	1.56924900	-2.21454900	H	2.23497600	0.95135700	0.80142000
C	5.16608200	-0.36477700	-1.11779000	C	5.49429100	1.35643100	-0.04046600
H	5.56574200	-0.82456400	0.94440500	H	6.64492900	-0.02643200	-1.22012600
H	4.51140000	0.25585200	-3.07115100	H	4.09533200	2.53378000	1.09336500
H	5.99848500	-0.95069100	-1.49454000	H	6.31303000	2.05764100	0.08609600
Gibbs free energy		-2300.149518 Hartree				-2300.147745 Hartree	

Energy difference accounts for 4.655 kJ mol^{-1} with the grey structure being more stable than the blue structure.

1. Bekker, H.; Van Den Berg, J. P.; Wassenaar, T. A. A Method to Obtain a Near-Minimal-Volume Molecular Simulation of a Macromolecule, Using Periodic Boundary Conditions and Rotational Constraints. *J. Comput. Chem.* **2004**, *25* (8), 1037–1046. https://doi.org/10.1002/jcc.20050.
2. Van Der Spoel, D.; Lindahl, E.; Hess, B.; Groenhof, G.; Mark, A. E.; Berendsen, H. J. C. GROMACS: Fast, Flexible, and Free. *J. Comput. Chem.* **2005**, *26* (16), 1701–1718. https://doi.org/10.1002/jcc.20291.
3. Kühne, T. D.; Iannuzzi, M.; Del Ben, M.; Rybkin, V. V.; Seewald, P.; Stein, F.; Laino, T.; Khaliullin, R. Z.; Schütt, O.; Schiffmann, F.; Golze, D.; Wilhelm, J.; Chulkov, S.; Bani-Hashemian, M. H.; Weber, V.; Borštnik, U.; Taillefumier, M.; Jakobovits, A. S.; Lazzaro, A.; Pabst, H.; Müller, T.; Schade, R.; Guidon, M.; Andermatt, S.; Holmberg, N.; Schenter, G. K.; Hehn, A.; Bussy, A.; Belleflamme, F.; Tabacchi, G.; Glöß, A.; Lass, M.; Bethune, I.; Mundy, C. J.; Plessl, C.; Watkins, M.; VandeVondele, J.; Krack, M.; Hutter, J. CP2K: An Electronic Structure and Molecular Dynamics Software Package - Quickstep: Efficient and Accurate Electronic Structure Calculations. *J. Chem. Phys.* **2020**, *152* (19), 194103. https://doi.org/10.1063/5.0007045.
4. Caldeweyher, E.; Bannwarth, C.; Grimme, S. Extension of the D3 Dispersion Coefficient Model. *J. Chem. Phys.* **2017**, *147* (3), 034112. https://doi.org/10.1063/1.4993215.

S9. Energetics of optimized structures

Free molecules

Species	E	ZPE	E_{therm}	S
**Acetone**	-193.017739	-192.934396	55.134	67.909
**Isopropanol**	-194.220408	-194.112590	71.073	71.062
**Phenylethanol**	-385.809660	-385.647817	106.737	89.775
**Acetophenone**	-384.609280	-384.471261	91.537	88.167

Reaction Intermediates

Species	E	ZPE	E_{therm}	S
**1**	-2109.146032	-2108.753841	264.445	175.715
**2NH1**	-2109.123505	-2108.738802	260.626	185.925
**2NH2**	-2109.123251	-2108.738498	260.677	187.186
**3RNH1**	-2300.716328	-2300.277196	296.940	201.063
**3SNH1**	-2300.717152	-2300.278	296.975	202.579
**3RNH2**	-2300.71801	-2300.278762	297.023	200.894
**3SNH2**	-2300.717372	-2300.277944	297.057	199.715
**4R**	-2300.741702	-2300.295834	300.548	194.043
**4S**	-2300.741606	-2300.297558	299.205	189.563
**5RNH1**	-2300.719687	-2300.277586	298.540	196.584
**5SNH1**	-2300.715741	-2300.274623	298.354	203.520
**5RNH2**	-2300.718828	-2300.276507	298.636	196.356
**5SNH2**	-2300.716779	-2300.274258	298.653	194.881
**6NH1**	-2109.124446	-2108.735878	262.615	182.163
**6NH2**	-2109.123315	-2108.734493	262.706	179.579

Transition States

Species	E	ZPE	E_{therm}	S
**$TS_{1\text{-}2NH1}$**	-2109.115708	-2108.723976	264.054	173.592
**$TS_{1\text{-}2NH2}$**	-2109.114982	-2108.729451	259.904	170.691
**$TS_{3R\text{-}4RNH1}$**	-2300.706041	-2300.267328	295.781	189.756
**$TS_{3S\text{-}4SNH1}$**	-2300.709784	-2300.270534	296.048	188.236
**$TS_{3R\text{-}4RNH2}$**	-2300.710441	-2300.270567	296.381	190.391
**$TS_{3S\text{-}4SNH2}$**	-2300.708135	-2300.269148	295.924	189.402
**$TS_{4R\text{-}5RNH1}$**	-2300.70675	-2300.264283	298.283	195.341
**$TS_{4S\text{-}5SNH1}$**	-2300.70858	-2300.267185	297.176	189.454
**$TS_{4R\text{-}5RNH2}$**	-2300.71663	-2300.274675	297.485	187.618
**$TS_{4S\text{-}5SNH2}$**	-2300.711211	-2300.269237	297.332	186.976
**$TS_{6NH1\text{-}1}$**	-2109.115537	-2108.727591	261.267	171.444
**$TS_{6NH2\text{-}1}$**	-2109.123642	-2108.735584	261.307	170.736

E: Electronic energy (PBE0/def2TZVP) [Hartree]
ZPE: Zero-point Energy [Hartree]
E_{therm}: Internal Thermal Energy [Kcal/Mol]
S: Entropy from translational degrees of freedom [Cal/Mol-Kelvin]
